# A global snapshot of the silent brain injury epidemic: A descriptive observational study of concussion coverage on YouTube

**DOI:** 10.34172/hpp.025.44339

**Published:** 2025-07-15

**Authors:** Aysha Jawed, Aryan Shabanpour, Nandita Gupta, Dennis Tudor, Yusuf Ghandi, Aria Mohebi

**Affiliations:** ^1^Department of Pediatrics, Johns Hopkins University School of Medicine, Johns Hopkins Children’s Center, Baltimore, MD, USA; ^2^Department of English, School of Public Health, University of Maryland, College Park, MD, USA; ^3^Biological Sciences Undergraduate Program, College of Computer, Mathematical, and Natural Sciences, University of Maryland, College Park, MD 20742, USA

**Keywords:** Concussion, Traumatic brain injury, YouTube, Social media, Health communication, Health education

## Abstract

**Background:**

Concussion is a prevalent form of traumatic brain injury worldwide with significant health consequences and far-reaching implications. Concussion protocols have been revised in recent years to protect athletes in increasing concussion reduction and timely treatment. Campaigns, news reports and mass media have also depicted coverage of concussions over the past decade especially in light of more athletes coming forward with long-term repercussions following repeated concussions.

**Methods:**

Previously, published studies have examined content pertaining to concussions across different social media platforms. Notably, the last comprehensive review of prevalent content across YouTube on concussions was ten years ago in 2014. Given updates in concussion protocols and clinical practice guidelines, increased news coverage and the movie release of Concussion in 2015, this study sought to examine and describe the sources, format and content covered among the top 100 widely viewed videos on YouTube a decade later.

**Results:**

Majority of the videos were posted by nongovernmental/organizational sources. Several testimonials by athletes on aftereffects of concussions were covered. Falls comprised the leading risk factor for concussions. Football, soccer, basketball and sailing represented the highest risk sports for concussions among the widely viewed videos. Many post-concussive symptoms were accounted for in the videos. Rest and activity limitations were featured as the leading treatments for concussions. Clinical, organizational, and health equity implications are presented.

**Conclusion:**

Recommendations to inform directions for patient and family education and clinical care on concussions are proposed.

## Introduction

 Concussions remain as one of the leading prevalent and longstanding forms of brain injury worldwide. A concussion is a mild traumatic brain injury that varies in recovery based on situational, contextual, and physiological factors.^1^ There is a wide range of post-concussive symptoms that can result and a range of treatments for concussions based on severity. Oftentimes, concussions are prominent across athletics and recreational activities.^2-4^ In recent years, there has been an uptake in reported consequences from the sequelae of concussions, especially in football.^5^

 Across the world, concussions have been observed on national television across many sports and on social media posts. Former football players have also shared their stories on developing neurodegenerative diseases from repeated head trauma following a longstanding series of concussions. Concussion protocols have been published by notable public health organizations including the National Institutes of Health, the Centers for Disease Control and Prevention and many healthcare systems worldwide.^6^ In addition, sports organizations have also developed guidelines to protect their athletes.^7^ Concussions have also received coverage on social media. Published studies have reviewed their coverage specifically across YouTube, TikTok, Twitter, Instagram, Pinterest, and Flickr as health communication mediums.^8-11^

 Notably, the last study of the most popular content across the concussions landscape on YouTube was conducted in 2014.^11^ A few years later, another study followed on concussion-related content specific to the adolescent population.^10^ Given that there have been increased revelations of neurodegenerative diseases linked with concussions in recent years,^12^ updates to concussion protocols and clinical practice guidelines, and the release of the movie Concussion starring Will Smith focused on investigating development of neurodegenerative disease among a former American football player, the present study sought to assess the current state of concussion content coverage on YouTube ten years later as the basis to inform more updated implications in regard to the reliability and clinical value of this content.

 Given the public accessibility of YouTube, it can be harnessed as not only a lay communication and knowledge dissemination tool but also as a global health communication medium. In addition based on a recent study conducted by the PEWS organization, YouTube continues to lead as the most widely accessed social media platforms in contemporary times.^13^ It follows that the goals of the present study are the following: 1) to describe sources, format and content of concussions across YouTube; 2) assess whether content covered is in line with current global concussion protocols and guidelines; and 3) present clinical care, organizational, and health equity implications and recommendations for future research and practice.

## Methods

 The research design of the present study was both descriptive and cross-sectional in nature. Observational data was collected at one conceptual point in time from the YouTube social media platform. In July 2024, browser history on the computers of six authors was cleared. The researchers conducted a search on YouTube using specific keywords and descriptors in both singular and plural forms pertaining to concussions. Piloting various keywords (e.g. concussion, post-concussive, traumatic brain injury) guided the process in narrowing the key terms that yielded videos pertinent to concussions, highest view counts across videos, and greatest cumulative views for the top 30, 60, and 100 videos, respectively. Following completion of these pilot searches, the keyword that formed the search strategy was “concussion” in its singular form which ultimately yielded the most widely viewed videos surrounding concussions. The results were subsequently filtered by view count, and the URLs for the 100 most widely viewed videos were extracted and consolidated in a separate file. Any overlapping URLs were deleted and replaced. Solely one URL for each video was kept for coding and analysis.

 Next, the researchers created a codebook based on a comprehensive review of the literature and protocols from a wide range of authoritative and expert sources such as the American Academy of Pediatrics, American Neurological Association, Centers for Disease Control and Prevention, sports organizations, among more. Each researcher viewed and coded all videos during July 2024. Intra- and inter-rater reliability of the coding was demonstrated across researchers. The following information was reviewed and coded for each video in the sample: (a) source of upload, (b) format, (c) number of views, (d) length (in minutes), (e) year of upload, and (f) content. Given that the videos were publicly accessible, IRB approval was not required for this study.

###  Eligibility criteria 

 All of the videos reviewed were in English. Any videos that were not narrated nor presented written content in English were excluded from analysis. Content across all videos pertained to concussions. Each researcher viewed the full video, which represented the unit of analysis. No exclusionary criteria was applied to videos based on the length of time.

###  Measurements and coding specifications

 The instrument included the following basic information: coder, assigned video identification number, date of upload for the video, date of coding for the video, length of video (in minutes), number of views, and title of the video. Next, there were three sections for source of upload, format and content in the instrument. Content included multiple content categories consisting of many variables (reviewed below), all of which were coded dichotomously (yes or no) to indicate presence or absence across each video.

 The source of upload for each video was coded into one of the following four categories: organizational/nongovernmental, consumer, governmental, and other sources. The categories for coding format included Documentary; Interview; Demonstration/Experiment; Talk by Professional; TV Talk Show/Discussion panel; Animation; Still images; News report with Anchor; V-blog; Advertisement; Testimonial/Story; Movie Trailer/Scene, Multiple Formats; and Other Formats. The following 16 content categories were created in this codebook: (a) illness/mortality; (b) post-concussive symptoms; (c) trauma; (d) treatments; (e) risk factors; (f) surface of injury; (g) setting of injury; (h) healthcare utilization; (i) athletics; (j) policies and protocols; (k) developmental groups; (l) additional populations; (m) community engagement; (n) post-concussive testing; (o) health benefits; (p) social factors; and (q) open-ended comments on misinformation or disinformation depicted in the video. Conceptualization of the codebook involved creating these content categories to account for the comprehensive range of domains, dimensions, and determinants pertaining to concussions.

###  Demonstration of intra- and inter-rater reliability 

 The researchers demonstrated both inter- and intra-rater reliability of the data from coding content across all of the widely viewed videos in this sample. To demonstrate intra-rater reliability, the researchers utilized a random number generator to randomly select 10 videos and re-coded them within a couple of weeks of the original coding. This analysis included each dichotomously coded (Yes versus No) content variable in the instrument. Intra-rater reliability was found to be high (Kappa = 0.94). Inter-rater reliability was also demonstrated across coding completed by all of the researchers. A random number generator prompted selection of ten of the coded videos for analysis across each variable. Next, the researchers reviewed coding responses to clarify any discrepancies in the coding instrument. Inter-rater agreement was found to be high (Kappa = 0.93).

###  Statistical analysis 

 Composite statistical analyses were conducted in this study which involved computing descriptive statistics for the number of views, length, and different source, format, and content variables. In addition, analysis of observational data obtained for each video included computing frequencies and percentages of source, format, and content of each video. Across content categories, the number of videos that covered each content variable was first identified. Next, the total number of views from those videos reviewing the specific content variable was determined. The proportion of cumulative views for each content variable was subsequently determined by dividing the number of views culminated from this subset of videos by the total cumulative views across all 100 widely viewed videos in the sample (N = 189,126,703 views). This process of analysis was conducted for each variable across all content categories in the codebook. All data analyses were conducted using Excel and Statistical Package for the Social Sciences (SPSS).

## Results

 The total number of views for the sample of the 100 most widely viewed videos pertaining to concussions was 189 126 703. The view counts ranged from 73 862 to 29 496 012. These widely viewed videos were posted between 2008 to 2023. Length of videos ranged from 0.27 minutes to 113.92 minutes. The median length of the widely viewed videos was 2.67 minutes. The interquartile range for the sample ranged from 1.67 minutes to 4.57 minutes.

 Majority of the videos (N = 53) were posted by nongovernmental/organizational sources and accounted for greater than 110 million views and nearly 60% of the cumulative views. Other formats were represented in 38 videos, culminating in almost 68 million views and about 36% of the cumulative views. Still images were covered in 28 videos, generating nearly 43 million views and approximately 23% of the cumulative views. Notably, although testimonials were featured in a comparative number of videos (N = 26), the cumulative views were substantially higher (> 57 million) and accounted for ~30% of these cumulative views.

 Mortality was depicted in 11 videos, garnering more than 45 million views which consisted of almost 24% of the cumulative views. Traumatic brain injury was delineated in 23 videos, populating approximately 43 million views (~23% of the cumulative views). Chronic traumatic encephalopathy (CTE) was included in 12 videos, attracting nearly 30 million views which represented about 16% of the cumulative views. Similarly, repeated head trauma was covered in 12 videos; however, the cumulative views for these videos was smaller ( > 26 million) and comprised ~14% of the cumulative views. Neurodegenerative disease was featured in 8 videos, culminating in greater than 27 million views and comprised about 14% of the cumulative views. Choking the brain was presented in 3 videos, attracting more than 24 million views that yielded nearly 13% of the cumulative views.

 Loss of consciousness was covered in 28 videos, attracting approximately 54 million views (~29% of the cumulative views). Disorientation was delineated in 25 videos, generating almost 48 million views (~25% of the cumulative views). Short-term memory loss was presented in 22 videos, yielding nearly 44 million views and ~23% of the cumulative views. Headaches were featured in 21 videos, garnering nearly 33 million views and about 18% of the cumulative views. Vomiting was portrayed in 11 videos, culminating in almost 30 million views and comprising ~16% of the cumulative views. Dizziness was reviewed in 15 videos, populating nearly 24 million views (~13% of the cumulative views).

 Rest was covered in 25 videos, accounting for more than 46 million views and approximately 25% of the cumulative views. Activity limitations were featured in 18 videos, culminating in more than 44 million views and about 23% of the cumulative views. Relaxation techniques were reviewed in 2 videos, garnering greater than 30 million views (about 16% of the cumulative views). Meditation was delineated in 1 video and generated more than 29 million views (about 16% of the cumulative views). Emergency departments were portrayed in 8 videos, culminating in approximately 21 million views (~11% of the cumulative views). Hospitalization was depicted in 7 videos, accounting for almost 19 million views (approximately 10% of the cumulative views).

 Falls were integrated in 53 videos, generating approximately 80 million views ( > 40% of the cumulative views). Collisions were featured in 22 videos, accounting for > 33 million views (~18% of the cumulative views). Fields were presented in 32 videos, culminating in almost 76 million views which comprised almost 40% of the cumulative views. Grass was portrayed in 34 videos, attracting nearly 71 million views (~37% of the cumulative views).

 Overall, contact sports were reviewed among 40 videos, attracting more than 60 million views (~32% of the cumulative views). Football was covered in 37 videos, garnering greater than 58 million views (about 30% of the cumulative views). Soccer was featured in 14 videos, generating almost 23 million views (about 12% of the cumulative views). Sailing was depicted in 2 videos, garnering almost 17 million views (nearly 9% of the cumulative views). Basketball was presented in 10 videos, yielding more than 11 million views (approximately 6% of the cumulative views).

 Young adults were featured in 12 videos, attracting almost 46 million views (~24% of the cumulative views). Sports organizations were covered in 14 videos, culminating in greater than 30 million views (~16% of the cumulative views). Athletes were depicted in 39 videos, accounting for almost 68 million views and ~36% of the cumulative views. Concussion protocols were reviewed in 6 videos, garnering more than 11 million views (~6% of the cumulative views). Testing with asking questions was presented in 5 videos, accounting for more than 19 million views (about 10% of the cumulative views). Tables 1-6 present a comprehensive breakdown of descriptive statistics for source, format and content covered across the widely viewed videos on concussions across YouTube.

**Table 1:** Frequencies, view counts, and cumulative view count percent of widely viewed concussion videos by sources

**Sources**	**N**	**View count **	**Cumulative view count percent (%)**
Nongovernmental/Organizational	53	110,736,626	58.55
Other	15	39,077,048	20.66
Consumer	28	37,962,117	20.07
Governmental	4	1,350,912	0.71

**Table 2:** Frequencies, view counts, and cumulative view count percent of widely viewed concussion videos by format

**Format**	**N**	**View count **	**Cumulative view count percent (%)**
Other formats	38	67,879,583	35.84
Testimonial	26	57,477,284	30.39
Still images	28	42,817,096	22.64
Movie trailer/scene	4	34,852,599	18.43
News report with anchor	13	32,446,639	17.16
Documentary	7	30,299,132	16.02
Demonstration/experiment	22	23,882,875	12.63
V-Blog	6	22,679,019	11.99
Interview	6	20,328,720	10.75
Animation	23	20,232,394	10.7
TV talk show/discussion panel	4	14,827,251	7.84
Talk by professional	26	14,544,128	7.69
Multiple formats	6	3,881,593	2.05
Advertisement	4	2,259,284	1.19

More than one response is possible across videos.

**Table 3:** Frequencies, view counts, and cumulative view count percent of widely viewed concussion videos by trauma

**Trauma **	**N**	**View count **	**Cumulative view count percent (%)**
Fall	53	80,160,787	42.38
Collision	22	33,580,251	17.76
Struck by individual	24	21,212,104	11.22
Struck by object	8	18,987,268	10.04
Motor vehicle accident	7	8,262,216	4.37
Bicycle accident	2	2,068,234	1.09
Assault/fight	2	1,871,585	1
Physical abuse	1	198,539	0.1

More than one response is possible across videos.

**Table 4:** Frequencies, view counts, and cumulative view count percent of widely viewed concussion videos by illness/mortality

**Illness/Mortality**	**N**	**View count **	**Cumulative view count percent (%)**
Mortality	11	45,200,109	23.9
Traumatic brain injury	23	43,171,284	22.83
Chronic traumatic encephalopathy	12	29,565,439	15.63
Neurodegenerative disease	8	27,209,790	14.39
Repeated head trauma	12	26,230,910	13.87
Choking the brain	3	24,149,214	12.77
Mood/mental health disorders	16	13,417,865	7.09
Depression	11	12,702,784	6.72
Dementia	6	6,053,219	3.2
Anxiety	6	3,730,235	1.97
Brain bleed (subdural hematoma)	5	3,699,478	1.96
Skull fracture	8	3,310,481	1.75
Seizures/convulsions	6	2,185,790	1.16
Brain bruise/contusion	3	729,593	0.39
Paralysis	2	394,534	0.21
ADHD	1	348,288	0.18
Disability	1	163,754	0.09

More than one response is possible across videos.

**Table 5:** Frequencies, view counts, and cumulative view count percent of widely viewed concussion videos by post-concussive symptoms

**Post-concussive symptoms **	**N**	**View count **	**Cumulative view count percent (%)**
Loss of consciousness	28	53,908,906	28.5
Disorientation	25	47,562,095	25.15
Short-term memory loss	22	43,980,739	23.25
Headache	21	33,051,836	17.48
Vomiting	11	29,771,606	15.74
Dizziness	15	23,686,358	12.52
Nausea	7	18,417,436	9.74
Bleeding	6	18,273,110	9.66
Unaware	5	17,416,193	9.21
Difficulty breathing	3	16,915,118	8.94
Responding slowly	4	16,277,853	8.61
Cognitive issues	11	15,669,199	8.29
Difficulty focusing	12	12,987,111	6.87
Long-term memory loss	7	11,112,241	5.88
Social Inhibition	1	9,867,268	5.22
Fatigue	13	9,243,183	4.89
Blurred vision	9	7,618,657	4.03
Difficulty sleeping	7	5,916,181	3.13
Slurred speech	8	5,469,427	2.89
Pain to other organs	5	4,802,619	2.54
Lightheadedness	4	4,689,049	2.48
Anger/aggression	8	4,511,813	2.39
Lost balance	9	3,974,390	2.1
Difficulty thinking	4	3,910,868	2.07
Light sensitivity	11	3,635,969	1.92
Learning difficulties	3	2,744,219	1.45
Chronic pain	1	2,105,425	1.11
Nightmares	1	2,105,425	1.11
Sensory impairment	6	1,745,969	0.92
Brain fog	5	1,504,171	0.8
Vision impairment	2	1,339,677	0.71
Migraines	2	1,219,278	0.64
Irritability	4	1,017,418	0.54
Ringing in the ears	2	985,954	0.52
Trembling	1	972,848	0.51
Unequal pupils	2	721,774	0.38
Weakness in other organs	3	690,463	0.37
Poor muscle coordination	1	619,125	0.33
Combative	1	578,138	0.31
Fluid coming out of ears and nose	1	236,257	0.12
Missed days from school	1	149,854	0.08
Decrease in grades	1	143,295	0.08
Bedridden	1	135,245	0.07
Difficulty reading	1	125,854	0.07

More than one response is possible across videos.

**Table 6:** Frequencies, view counts, and cumulative view count percent of widely viewed concussion videos by treatments

**Treatments**	**N**	**View count **	**Cumulative view count percent (%)**
Rest	25	46,357,337	24.51
Activity limitations	18	44,091,177	23.31
Relaxation techniques	2	30,036,798	15.88
Meditation	1	29,496,012	15.6
Not Playing in game	8	9,085,156	4.8
Prescription medications	3	5,469,389	2.89
Ice	4	4,251,946	2.25
Low lit room	3	3,388,372	1.79
Observation at home	2	3,296,260	1.74
Rehabilitation	3	2,918,541	1.54
Monitor symptoms	4	1,458,290	0.77
Never playing sports again	3	1,249,763	0.66
Missing Season	4	896,222	0.47
Exercise	3	874,239	0.46
Over-the-counter medications	1	619,125	0.33
Avoid stimulation	3	564,955	0.3
Eye massage	1	540,786	0.29
Prevention	2	456,092	0.24
Nutrition	1	366,829	0.19
Stress reduction	1	200,283	0.11
Calisthenics	1	200,283	0.11
Yoga	1	200,283	0.11

More than one response is possible across videos

## Discussion

 As aforementioned, the top 100 widely viewed videos on concussions generated greater than 189 million views. There was an uptake in videos pertaining to concussions posted after 2015 following the release of the movie Concussions starring Will Smith. Many videos were posted by nongovernmental, consumer and other sources (e.g. a healthcare provider with a freestanding channel on YouTube). Several videos involved testimonials posted by former athletes, primarily in the NFL. Furthermore from an athletics perspective, concussions sustained from football were covered the most among sports followed by soccer, sailing, and basketball across the widely viewed videos. Falls were reviewed substantially and drew in increased engagement among the videos, likely also attributed to the fact that falls occur across all developmental and age groups. A wide range of illness and mortality related content was also presented in these videos especially pertaining to traumatic brain injury, CTE and neurodegenerative diseases attributed to concussions. In addition, there were many post-concussive symptoms included in the videos as well as a diversity of treatments based on severity of concussion. Rest and activity limitations were the widely recommended treatments for concussions across the videos. Healthcare utilization and developmental considerations for different populations were underrepresented among these videos.

 Notably, nongovernmental/organizational sources posted the majority of the widely viewed videos followed by other sources and consumers. Many of these nongovernmental/organizational sources included sports associations and news channels. Other sources included expert discussions as talks by professionals. Several of the consumer sources presented testimonials. Governmental sources in comparison generated less engagement across these videos. These findings suggest that it is possible that video resources published by the government may not be accessible or relatable to consumers. For example, the Centers for Disease Control and Prevention is a U.S. governmental organization that has published much concussion-related content online; notably only one video was published by the CDC in the widely viewed videos. Similarly, a different U.S. governmental organization, the National Institutes of Health (NIH) has taken an active role in concussion-based research and knowledge dissemination; however, none of the widely viewed videos were posted by the NIH. It follows that finding ways for organizations (e.g. associations, non-profit and for-profit foundations) and consumers to partner with the government could help create content that is more engaging and appealing and in turn could also support increased accessibility and visibility of content on concussions by the government. Furthermore, this collaboration could also create pathways to form expert panels that could be involved in the evaluation of future concussion-centered content on social media to formally assess reliability with clinical practice guidelines.

 Across athletics, football received the greatest coverage across the widely viewed videos. Similarly, testimonials by former NFL players on developing CTE as a sequalae of repeated head trauma from concussions were also widely featured. It is also significant to note that many of these videos were posted after 2015 following the release of the Concussion movie with Will Smith as the scientist in the lead role seeking to corroborate CTE from concussions sustained during football. In light of lawsuits and game rule changes implemented by the NFL, increased coverage of concussion protocols was also prevalent across the widely viewed videos. These videos heightened awareness on CTE and present several clinical care implications for both neurologists and radiologists in assessment of brain imaging in combination with neurological examination to inform diagnostic considerations for CTE among patients with repeated head trauma secondary to concussions. Testimonials and video footage of athletes on both collegiate and professional levels sustaining injuries during soccer and basketball also received substantial coverage in these videos. Notably although two videos covered concussions during sailing, their view count surpassed 16 million and brings to light implications for concussions in water-based sports. It follows that finding ways to disseminate knowledge acquired from these videos to relevant sports organizations, neurology clinics, and radiology centers could inform heightened awareness in engaging our community of practice to take an active role in connecting our lay population and athletes to timely and needed diagnostic evaluation and treatment following a concussion as well as in preventive practices for concussion reduction.

 It is imperative to note that the data is not yet compelling to make the case that social media is an evidence-based practice for concussion care. However, none of the widely viewed videos presented any misinformation, and several of them featured experts in science and healthcare presenting the mechanisms and treatment considerations for concussions. Furthermore, content on concussion protocols across these videos were reliable and integrated recommendations from guidelines on concussion care across both governmental and nongovernmental organizations worldwide (American Association of Neurological Surgeons, CDC, American Academy of Pediatrics, Academy of Neurologic Physical Therapy).^14^ Further evaluation of the credibility of these videos as part of patient and family education could further determine directions on their acceptability and efficacy in clinical practice for affected and at-risk patients. In addition, the number of views for the top 10 videos each surpassed 4 million views which could suggest that these videos are reaching patients more than our current systems in place (e.g. healthcare systems, schools, coaches/trainers, caregivers). Given both increased reliability and engagement of content across these videos, increased access to them across these systems could also be explored as a timely and efficient way in reaching these affected and high-risk patients across the community in heightening concussion reduction and treatment.

 Falls were also covered substantially across the widely viewed videos. Many fall protocols exist across healthcare systems and nursing homes.^15,16^ However, there was also scant coverage of healthcare utilization across the widely viewed videos which suggests that seeking treatment in a medical setting after sustaining a concussion may not draw in consumers as an acceptable strategy. In turn, fall protocols may be limited in reaching affected and at-risk patients given their increased visibility in healthcare spaces which were not well represented across the widely viewed videos. It follows that finding ways to increase dissemination of these videos on fall related considerations as part of concussion reduction and treatment for populations across the lifespan and engaging healthcare systems could increase accessibility to relatable content and support of seeking timely medical care. In turn, this could be another direction in heightening knowledge on falls as a risk factor for sustaining concussions.

 In addition from a health equity perspective although there was limited coverage of financial stressors in access to timely concussion care, a wide range of strategies were depicted for recovery following a concussion. For uninsured and underinsured patients with limited or no access to primary or subspecialty care, many of the options presented for treatment involve no cost and could be a point of education in the meantime until there is access to care in the future. For example, greater than 40 post-concussive symptoms ranging in severity were covered across the widely viewed videos and among treatments, rest and activity limitations generated the highest engagement via view counts. It follows that finding ways to increase ease of access to these videos for this population in the meantime could provide symptom relief and treatment in less severe cases of concussions.

 There were several limitations in the present study. First, the sample of videos was cross-sectional in nature; videos were extracted at one conceptual point in time for review. Given that view counts, emergence and sequence of videos are continually changing across social media, this limits replicability of this study. Interpretation of memory loss was decided by consensus of the research team to include content covering selective memory loss (e.g. could not remember circumstances surrounding concussion but everything else) which could certainly impact the interpretations of the findings from this study in regard to this construct. In addition, there could be possible sampling bias present given that that the search algorithm is unclear and further how it was utilized to populate the videos for this study. Also, the videos reviewed were in English which could preclude relevant videos with high engagement in different primary languages. View count was utilized as the metric in this study to assessment engagement among the widely viewed videos. However, it is impossible to know whether these videos were partially or fully viewed as well as any information about the viewers and whether any information was retained by the viewers. Future studies could also assess different engagement metrics (e.g. likes, tweets, shares, comments) along with implementing different scoring metrics (e.g. DISCERN tool) to analyze quality of content. Nevertheless, findings from this study present an abundance of information on the visibility of concussions worldwide and a wide range of strategies for concussion reduction and treatment since the last content analysis of the top 100 widely viewed videos on concussions published ten years ago.^11^

## Conclusion

 Concussions remain a leading brain injury epidemic across the world. Ten years later, content on concussions has expanded across both traditional and nontraditional media sources as seen in increased coverage of concussions in movies and TV shows. From a health equity perspective, social media also presents a range of treatments based on concussion severity that could be a helpline for underinsured and uninsured patients as well as for the rest of the lay population. Fortunately, concussion related content on social media across the sample of videos reviewed was in alignment with concussion protocols and clinical practice guidelines for concussion care and treatment, thereby heightening reliability. This finding further provides an opportunity to assess the credibility and efficacy of social media not only as a health communication medium on concussions in informing directions for patient and family education but also as an intervention for concussion prevention and reduction across our global population.

## Competing Interests

 The authors have no competing interests to declare.

## Ethical Approval

 This article does not contain any studies with human participants or animals performed by any of the authors.
